# Progress in Carnot and Chambadal Modeling of Thermomechanical Engine by Considering Entropy Production and Heat Transfer Entropy

**DOI:** 10.3390/e21121232

**Published:** 2019-12-16

**Authors:** Michel Feidt, Monica Costea

**Affiliations:** 1 Laboratory of Energetics, Theoretical and Applied Mechanics (LEMTA), URA CNRS 7563, University of Lorraine, 54518 Vandoeuvre-lès-Nancy, France; michel.feidt@univ-lorraine.fr; 2 Department of Engineering Thermodynamics, University POLITEHNICA of Bucharest, 060042 Bucharest, Romania

**Keywords:** Carnot engine, optimization, heat transfer entropy, entropy production, new efficiency limits

## Abstract

Nowadays the importance of thermomechanical engines in recognized worldwide. Since the industrial revolution, physicists and engineers have sought to maximize the efficiency of these machines, but also the mechanical energy or the power output of the engine, as we have recently found. The optimization procedure applied in many works in the literature focuses on considering new objective functions including economic and environmental criteria (i.e., ECOP ecological coefficient of performance). The debate here is oriented more towards fundamental aspects. It is known that the maximum of the power output is not obtained under the same conditions as the maximum of efficiency. This is shown, among other things, by the so-called nice radical that accounts for efficiency at maximum power, most often for the endoreversible configuration. We propose here to enrich the model and the debate by emphasizing the fundamental role of the heat transfer entropy together with the production of entropy, accounting for the external or internal irreversibilities of the converter. This original modeling to our knowledge, leads to new and more general results that are reported here. The main consequences of the approach are emphasized, and new limits of the efficiency at maximum energy or power output are obtained.

## 1. Introduction

The origin of the industrial era in the 19th century is strongly connected to thermomechanical engines whose importance in our society is well-known. Their rapid development and evolution have contributed to the foundation of thermodynamics as a new branch of science, mainly concerned with fundamental aspects, as well as numerous applications. 

The famous reference case is relative to the Carnot engine [1], but it refers only to an Equilibrium Thermodynamics modelling of a totally reversible engine. Additionally, the work done by Carnot generalizes the concept of efficiency according to the First Law of Thermodynamics starting from a purely mechanical approach [2], and the concept of the Carnot cycle. The interactions between the working fluid and the surroundings during a cycle consist of a mechanical energy exchange, *W*, and a calorific energy exchange, *Q*, which are of infinite time duration for reversible conditions. This leads to a quasi-static cycle, whose mean power output of the engine, $\dot{W}$, is zero. 

Since (approximately) the 80s, a new approach has been developed including the existence of unavoidable heat transfer irreversibilities (endo-reversible machine [3]), and afterward, internal irreversibilities of the converter were also considered, but less frequently. To take account of these irreversibilities, two main forms are reported in the literature:

- an entropic ratio, *I* [4];
- a production of entropy, that for a cycle is represented as a parameter, Δ*S_I_* [5].

In the more general case, the entropic ratio could be a function that needs to be specified. This will be considered in this paper.

The main objective encountered in the literature relative to the Non-Equilibrium Thermodynamics approach remains the power output optimization of the engine and the associated conditions (vector of state), as well as the efficiency associated with maximum power.

Numerous studies are concerned with the influence of:

- the form of the heat transfer laws at source and sink [6,7,8,9,10];
- the nature of the sources and sinks (thermostats, fluid flows without phase change) [11,12];
- various objective functions [10,13,14,15,16];
- consideration of added constraints [17,18,19,20,21,22,23,24,25];
- thermal losses or adiabaticity [26,27,28];
- various irreversibilities (mainly global approaches by considering Δ*S_I_* or *I* [4]; or introduced by mechanisms, namely solid or fluid energy dissipation) [5].

The present paper proposes a new modelling of an irreversible cycle focused on a necessary condition of the cycle existence, namely the heat transfer entropy. As the most important one for the cycle is the entering heat transfer entropy associated with the heat input in the converter (engine), it will be called hereafter, reference entropy, Δ*S*. According to the modeling assumptions that consider all cycle processes in the converter as irreversible, the total entropy production over a cycle, Δ*S_I_* is added to this reference heat transfer entropy. The coupling consequence of these two entropies in the analytical analysis of an irreversible cycle leads to new results that are reported here. 

This new modelling is developed for the exo-reversible and endo-irreversible Carnot engine configuration, as the first possible extension of the primitive Carnot cycle approach.

Then, the remodeled Chambadal [29] engine configuration is described by adding the influence of the converter internal irreversibility. The optimization approach considers two ways to introduce the converter internal irreversibility, namely the entropic ratio model and the entropic production one, each of them being developed for different dependence functions of the system parameters. The consequences of this addition on the optimized power output are reported, and the limit cases—the endo-reversible Carnot engine and endo-irreversible model—are shown to be recovered.

A discussion of the overall results and an attempt to generalize the present study provide new and important upperbounds for the engine performance. 

An ongoing next step will be the application of the same methodology to Curzon-Ahlborn [30] modelling of the Carnot engine by including internal irreversibilities of the converter and considering the importance of the heat transfer entropy.

## 2. The Endo-Irreversible Carnot Engine

### 2.1. Cycle Representation in T-S Diagram

**Figure 1:**
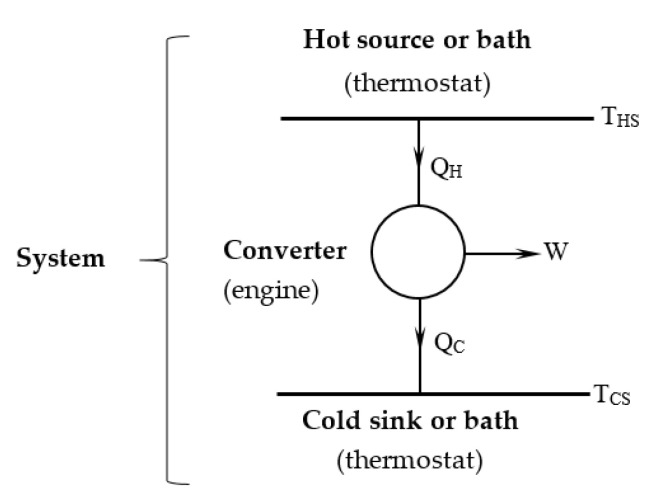
Schematic representation of the thermo-mechanical engine.

The thermomechanical engine is considered as a system composed of a hot bath (heat source), converter (engine) and cold bath (heat sink), as shown in Figure 1. The coupling of the three subsystems is made by the heat transferred from the source to the working fluid in the engine at its hot side, and the heat rejected by the working fluid to the sink at the cold side, respectively.

In the Carnot engine modeling, the source and sink are two infinite bath (thermostats) at temperatures *T_HS_* and *T_CS_* respectively. The Carnot engine operates upon an irreversible cycle with perfect thermal contact (no temperature difference) with the two bathes. Thus, one can call it an exo-reversible and endo-irreversible engine.

**Figure 2:**
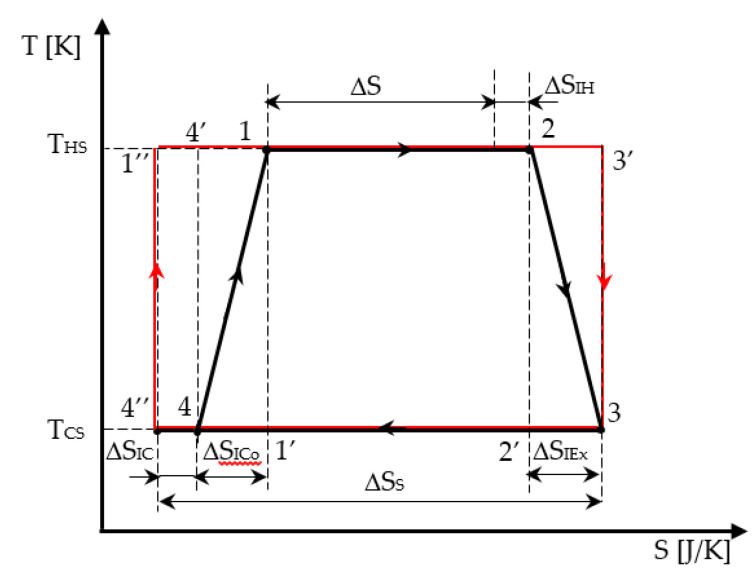
Representation of the Carnot engine cycle with internal irreversibility and the corresponding reversible one (in red) in T-S diagram.

The irreversible cycle is identified in Figure 2 from state 1 (beginning of the high temperature isotherm), 2 (exit of the high temperature isotherm and beginning of the adiabatic expansion), 3 (end of the adiabatic expansion and beginning of the low temperature isotherm), and 4 (exit of the low temperature isotherm and beginning of the adiabatic compression, up to state 1). The reversible cycle (marked in red) is also represented in Figure 2 as a starting graphical cycle helping to emphasize the effect of each internal irreversibility considered in the modeling.

Both adiabatic and isothermal transformations of the cycle are considered irreversible. Thus, the irreversibility on the isothermal processes is represented on the horizontal lines 12 and 34 by the corresponding production of entropy. As the irreversibility on the adiabatic processes in the cycle is also emphasized by entropy production, the graphical representation is associated to a polytropic processes represented in the T-S diagram by a corresponding dependence *T = f(S)*. For simplicity, a linear dependence *T = f(S)* between states 2 and 3, and 4 and 1, respectively, replaced the real one in the analytical and graphical analysis of the cycle.

Consequently, the production of entropy on a cycle Δ*S_I_* representing the total amount of entropy produced in the universe per cycle (= engine + environment) is expressed as:

(1) $$\Delta S_{I}=\Delta S_{IH}+\Delta S_{IEx}+\Delta S_{ICo}+\Delta S_{IC}$$

with Δ*S_IH_,* production of entropy on the hot isotherm;

Δ*S_IEx_,* production of entropy during the adiabatic expansion;
Δ*S_ICo_,* production of entropy during the adiabatic compression; and
Δ*S_IC_,* production of entropy on the cold isotherm, as shown in Figure 2.

Furthermore, for the hot temperature isotherm, the entropy production Δ*S_IH_* corresponds to:

(2) $$\Delta S_{H}=\Delta S+\Delta S_{IH},$$

where the reference heat transfer entropy is expressed as:

(3) $$\Delta S=\frac{Q_{H}}{T_{HS}},$$

and it equals the entropy change in the hot bath (in module).

Similarly, for the cold temperature isotherm, the entropy production Δ*S_IC_* corresponds to:

(4) $$\Delta S_{C}=\Delta S_{S}-\Delta S_{IC},$$

as all production of entropy terms are positive (see Figure 2).

**Figure 3:**
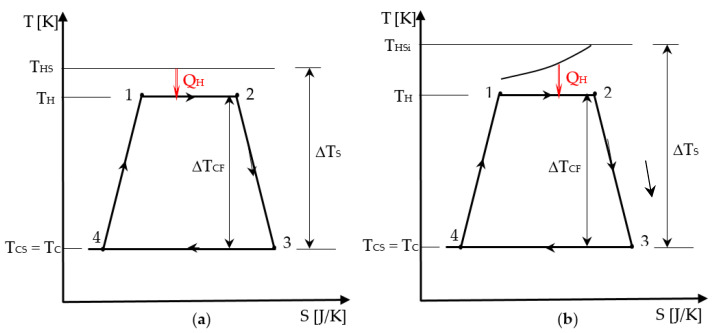
Representation of the associated cycle to the Chambadal engine in T-S diagram: (**a**) source of constant temperature; (**b**) source of finite heat capacity.

The two entropy productions Δ*S_IH_* and Δ*S_IC_* correspond to the graphical representation in Figure 3.

One outlines here that the entropy balance over the irreversible cycle is expressed as:

(5) $$\Delta S+\Delta S_{I}=\Delta S_{S}=\frac{Q_{C}}{T_{CS}},$$

where Δ*S_S_* represents the heat transfer entropy corresponding to the heat transferred to the sink.

Note that the commutations between isothermal and adiabatic processes are assumed to be instantaneous. Moreover, one observes that while the two real isothermal processes are well identified on the diagram, it is not the same for the irreversible adiabatic processes linking state 2 to 3, and then 4 to 1. All the curves inscribed in the rectangle (2-3′-3-2′-2) satisfying the criterion of increasing entropy on the adiabatic expansion process are possible.

Plotting a linear transformation in the *T-S* diagram between state 2 and 3 is not the only physical acceptable solution, because in the frame of Non-Equilibrium Thermodynamics, one only has access to points 2 and 3 representing the adiabatic case.

The same discourse can be renewed for the adiabatic compression between 4 and 1. In short, all the curves inscribed in the rectangle (4-4′-1-1′-4) satisfying the criterion of increasing entropy on the adiabatic process are possible. The linear transformation in the *T-S* diagram between state 4 and 1 is again a possible intermediate solution.

This irreversibility approach is similar to the one of a recent publication [31].

The graphical analysis of the cycle with linearized processes between 23 and 41 illustrated in Figure 2 shows the area representing the corresponding linearized work per cycle *W_L_* as:

(6) $$W_{L}=\left(\frac{\Delta S_{H}+\Delta S_{C}}{2}\right)\left(T_{HS}-T_{CS}\right).$$

This ‘geometric’ work (6) is different from the ‘thermodynamic’ (or mechanical) work that will be expressed in Section 2.2 by (10) since (6) depends on the linear assumption regarding the representation of the irreversible adiabatic processes between 23 and 41 as done in many references [31]. Nevertheless, it does not reflect the exact physical behavior of the endo-irreversible Carnot heat engine that could be different to the linear one. Thus, only (10), which is based on energy and entropy balances, will provide the correct expression of the mechanical work.

### 2.2. Analytical Model

Considering the adiabaticity (no thermal loss) and exo-reversibility assumptions for the cycle in this model, the heat transfer at the source and sink are respectively:

(7) $$Q_{HS}=Q_{H}>0\;\;;\;\;Q_{CS}=Q_{C}<0.$$

The exo-reversibility of the heat transfer relative to the two thermostats imposes:

(8) $$T_{HS}=T_{H}\;\;;\;\;T_{CS}=T_{C}.$$

Thus, the entropy balance on the system can be written as:

(9) $$\frac{Q_{HS}}{T_{HS}}+\Delta S_{I}=\frac{\left|Q_{CS}\right|}{T_{CS}},$$

where from the expression of the mechanical work per cycle from the First Law when the entropy variation corresponding to the heat transfer converted at the source is considered as reference heat transfer entropy, Δ*S*, $\Delta S_{H}=\Delta S$. becomes:

(10) $$W=Q_{HS}-\left|Q_{CS}\right|=\left(T_{HS}-T_{CS}\right)\Delta S-T_{CS}\Delta S_{I}.$$

The first term of the second equality represents the reversible work, and the second one, the mechanical dissipation (by fluid and solid friction mainly).

The expression of *W* in (10) marks the presence of the internal entropy production over a cycle Δ*S_I_* that is supposed as a parameter. Its relationship with the ratio method is introduced below, by emphasizing Δ*S* in (10):

(11) $$W=\Delta S\left[\left(T_{HS}-T_{CS}\right)-T_{CS}\frac{\Delta S_{I}}{\Delta S}\right].$$

Thus, the ratio Δ*S_I_*/Δ*S* appears in (11) and it corresponds to the irreversibility degree *d_I_* introduced by Novikov [32]. This parameter is also related to an entropic ratio according to Ibrahim’s approach [4], which represents the absolute value of the ratio of the entropy change in the cold bath to the entropy change in the hot bath and is given as:

(12) $$I=\frac{\Delta S_{S}}{\Delta S}.$$

One notes that this ratio can be particularized (i.e., *I_H_ =* Δ*S_S_*/Δ*S_H_*), since it represents a coefficient relative to an entropy variation corresponding to the heat exchanges at the hot and cold sides of the converter. Hence, other assignments could be given (*I_C_*, *I_HS_*, *I_CS_*). For the adiabatic exo-reversible Carnot engine, this ratio is *I* = *I_H_* = *I_HS_* and moreover, it is easy to show that:

(13) $$I=1+d_{I}=1+\frac{\Delta S_{I}}{\Delta S},$$

and

(14) $$W=\Delta S\left[\left(T_{HS}-T_{CS}\right)-T_{CS}\left(I-1\right)\right]=\Delta S[T_{HS}-I\cdot T_{CS}],$$

(15) $$\eta_{I}=\frac{W}{Q_{HS}}=1-I\frac{T_{CS}}{T_{HS}}.$$

It results that the internal irreversibility diminishes the mechanical work and cycle efficiency.

It remains to analyze how *I* or Δ*S_I_* vary with the cycle quantities. It can be reasonably assumed that Δ*S_I_* and (or) *I* depend on Δ*S* and (or) Δ*T_S_ = T_HS_ – T_CS_*, as the two temperatures are generally parameters of the problem for the Carnot endo-irreversible engine. Hence, two functions can be proposed, whose general forms (currently unknown in practice) are written as:

(16) $$\Delta S_{I}=f_{SI}\left(\Delta T_{S},\Delta S\right)\;\;;\;\;I=f_{I}\left(\Delta T_{S},\Delta S\right).$$

If there is an optimum of *W*, it must satisfy the necessary condition imposed by one of the following equation sets:

(17) $$\frac{\partial W}{\partial \Delta T_{S}}=\Delta S-T_{CS}\frac{\partial f_{SI}}{\partial \Delta T_{S}}=0\;\;;\;\;\frac{\partial W}{\partial \Delta T_{S}}=\Delta S\left(1-\frac{\partial f_{I}}{\partial \Delta T_{S}}\right)=0,$$

(18) $$\frac{\partial W}{\partial \Delta S}=\Delta T_{S}-T_{CS}\frac{\partial f_{SI}}{\partial \Delta S}=0\;\;;\;\;\frac{\partial W}{\partial \Delta S}=\Delta T_{S}-T_{CS}\frac{\partial f_{I}}{\partial \Delta S}=0.$$

## 3. The Chambadal Engine

The representation of the Chambadal engine irreversible cycle in Figure 3 shows that in addition to the internal irreversibility of the cycle that was graphically detailed in Section 2.1, the external irreversibility corresponding to the heat transfer at temperature difference at the hot side appears. Since the same components of the internal irreversibility for the endo-irreversible Carnot engine are considered here as well, the cycle representation was simplified by only preserving its shape, and the new irreversibility completing the model was emphasized. Hence, in Figure 3 case (a) corresponds to a heat transfer from a source of constant temperature *T_HS_*, while case (b) shows a finite heat capacity source with the inlet temperature *T_HSi_*. The temperature difference of the source and sink level, Δ*T_S_*, compared to the cycled fluid one, Δ*T_CF_*, that remains the same, is also relevant for the heat input in the two cases.

Note that the external irreversibility at the sink is neglected in this approach by considering the heat rejection to sink occurring at ambient temperature (*T_C_ = T_CS_ = T_0_*).

The Chambadal engine model is concerned with steady-state operation of the engine and consequently, heat rates and power output are used. We propose here to convert this modeling with reference to a cycle and the related energies-heat and work, thus leading to a different model, the quasi-Chambadal engine.

It involves a thermostat or source of finite heat capacity rate ${\dot{C}}_{H}={\dot{m}}_{H}c_{p}{}_{H}$, with an input temperature *T_HSi_*. 

Hence, when the source is a thermostat of constant temperature, *T_HS_*, the heat transfer at the hot end is expressed as:

(19) $${\dot{Q}}_{H}=U_{H}A_{H}\left(T_{HS}-T_{H}\right),$$

with *K_H_ = U_H_* · *A_H_*, heat transfer conductance of the source corresponding to the steady state operation regime.

When the source has a finite heat capacity, it involves a temperature variation of the primary fluid in the corresponding heat exchanger. As the input temperature of the primary fluid *T_HSi_* is generally known, by using the effectiveness *ε* – number of heat transfer units *NTU* method [27], one gets:

(20) $${\dot{Q}}_{H}=\varepsilon_{H}{\dot{C}}_{H}\left(T_{HSi}-T_{H}\right).$$

Note that the two expressions of ${\dot{Q}}_{H}$ averaged on a time duration Δ*t*, allow to recover the caloric energy *Q_H_*, or the mass specific energy (often used) that results by dividing ${\dot{Q}}_{H}$ with the mass flow rate of the cycled fluid. Thus, a general expression of the heat transferred at the source is:

(21) $$Q_{H}=G_{H}\left(T_{HSi}-T_{H}\right)=T_{H}\cdot \Delta S,$$

where *G_H_* generally represents a finite physical dimension of the source (thus also of the system) with reference to energy here, its unit being J/K.

### 3.1. Optimization Approach for the Entropic Ratio Model

The previous equation is completed in this approach by the entropy balance expressed over a cycle as:

(22) $$I_{H}\frac{Q_{H}}{T_{H}}=\frac{Q_{C}}{T_{0}}.$$

This leads to the expression of the mechanical work output as:

(23) $$W=G_{H}\left(T_{HSi}-T_{H}\right)\left(1-I_{H}\frac{T_{0}}{T_{H}}\right).$$

By taking into account that *I_H_* is a potential function of *T_H_*, the mechanical work maximum value *MaxW* search should satisfy the necessary condition:

(24) $$\frac{\partial W}{\partial T_{H}}=0=-1+I_{H}\frac{T_{0}}{T_{H}}-\left(T_{HSi}-T_{H}\right)\frac{I_{H,H\;}T_{0}\;T_{H}-I_{H}T_{0}\;}{T_{H}{}^{2}},$$

where $I_{H,H}=\frac{\partial I_{H}\;}{\partial T_{H}}$.

The term rearrangement leads to an equation of second degree or more to be solved:

(25) $$\left(T_{HSi}-T_{H}\right)T_{0}\;T_{H}I_{H,H\;}=I_{H}T_{0}T_{HSi}-T_{H}{}^{2}.$$

Possible cases are:

(i) *I_H_* is a constant parameter.

This implies $\partial I_{H}/\partial T_{H}=I_{H,H\;}=0$.

Thereby, the optimum temperature at the hot-end, *MaxW* and the corresponding cycle efficiency respectively, result as:

(26) $$T_{H}{}^{*}=\sqrt{I_{H}T_{0\;}T_{HSi\;}}$$

(27) $$MaxW=G_{H}{\left(\sqrt{T_{HSi}}-\sqrt{I_{H}T_{0\;}}\right)}^{2},$$

(28) $$\eta_{I}\left(MaxW\right)=1-\sqrt{\frac{I_{H}T_{0\;}}{T_{HSi}}}$$

It is obvious that the rise of *I_H_* parameter results in an increase of the optimal temperature $T_{H}{}^{*}$ and has the opposite effect (decrease) on *MaxW* and the associated cycle efficiency.

(ii) *I_H_* is a linear function of Δ*T_T_* = *T_H_* – *T_0_* (see Figure 3), with values ≥ 1.

One considers the following expression for *I_H_*:

(29) $$I_{H}=C_{I}\left(T_{H}-T_{0}\right)+1,\;\;\;with\;\;\;\;C_{I}=I_{H,H\;}$$

By combining (25) with (29), one gets:

(30) $$C_{I\;}\left(T_{HSi}-T_{H}\right)T_{0}\;T_{H}=\left[C_{I\;}\left(T_{H}-T_{0}\right)+1\right]T_{0}T_{HSi}-T_{H}{}^{2}.$$

The above equation leads to the expression of the optimum temperature of the cycled fluid at the hot side expressed by:

(31) $$T_{H}{}^{*}=\sqrt{T_{0\;}T_{HSi\;}}\;.$$

It results that irreversibility does not affect the temperature $T_{H}{}^{*}$, but it does affect the mechanical work and the cycle efficiency, whose expressions become:

(32) $$MaxW=G_{H}\left(1-C_{I}T_{0\;}\right){\left(\sqrt{T_{HSi}}-\sqrt{T_{0\;}}\right)}^{2},$$

(33) $$\eta_{I}\left(MaxW\right)=\left(1-C_{I}T_{0\;}\right)\left(1-\sqrt{\frac{T_{0\;}}{T_{HSi}}}\right).$$

The comparison of (27) and (32) clearly shows the maximum work decrease in case ii) and by a completely different expression. The same happens for the efficiency at maximum work, when comparing (28) and (33).

### 3.2. Optimization Approach for the Entropic Production Model

The entropy balance takes a similar form to that of Section 2.2, namely:

(34) $$\frac{Q_{H}}{T_{H}}+\Delta S_{I}=\frac{Q_{C}}{T_{C}},$$

with *T_C_* = *T_CS_ = T*_0_.

One notes that the production of entropy on the cycle in (34) is not the same with (1) in the endo-irreversible Carnot engine case, since it also considers the production of entropy corresponding to the heat transfer at finite temperature difference (see Figure 3).

The mechanical work is expressed as:

(35) $$W=Q_{H}\left(1-\frac{T_{CS}}{T_{H}}\right)-T_{CS}\Delta S_{I}.$$

By using (21) in (35), one gets:

(36) $$W=G_{H}\left(T_{HSi}-T_{H}\right)\left(1-\frac{T_{CS}}{T_{H}}\right)-T_{CS}\Delta S_{I}.$$

The term Δ*S_I_* is a potential function of *T_H_* (also increasing one). Therefore, the *MaxW* search should satisfy the necessary condition:

(37) $$\frac{\partial W}{\partial T_{H}}=0=-\left(1-\frac{T_{CS}}{T_{H}}\right)+\left(T_{HSi}-T_{H}\right)\frac{T_{CS}}{T_{H}{}^{2}}-\frac{T_{CS}\Delta S_{I,H}}{G_{H}}=0.$$

This form is simpler than the previous one, providing an equation of the second degree or more to solve.

The possible cases are as follows:

(i) Δ*S_I_* is a constant parameter.

This implies $\partial S_{I}/\partial T_{H}=\Delta S_{I,H}=0$, which delivers:

(38) $$T_{H}{}^{*}=\sqrt{T_{HSi\;}T_{CS\;}}\;.$$

The expressions of *MaxW* and $\eta_{I}\left(MaxW\right)$ are straightforward:

(39) $$MaxW=G_{H}{\left(\sqrt{T_{HSi}}-\sqrt{T_{CS}}\right)}^{2}-T_{CS}\Delta S_{I},$$

(40) $$\eta_{I}\left(MaxW\right)=1-\sqrt[{}]{\frac{T_{CS\;}}{T_{HSi}}}\left(1+\frac{\Delta S_{I}}{\Delta S}\right).$$

(ii) A linear dependence of Δ*S_I_* on the temperature difference Δ*T_T_* = *T_H_* – *T_CS_* is considered.

We assume that:

Δ*S_I_* = 0  if Δ*T_T_* = 0;

Δ*S_I_* = Δ*S_IS_* (new parameter) if Δ*T_T_* = Δ*T_S_* = *T_HSi_* – *T_CS_* (see Figure 3b),

where from:

(41) $$\Delta S_{I}=\Delta S_{IS}\frac{\Delta T_{T}}{\Delta T_{S}}=\Delta S_{IS}\frac{T_{H}-T_{CS}}{T_{HSi}-T_{CS}}=C_{IS}\left(T_{H}-T_{CS}\right).$$

The mechanical work optimization imposes:

(42) $$\frac{\partial W}{\partial T_{H}}=0=G_{H}\left(\frac{T_{HSi}\;T_{CS}}{T_{H}{}^{2}}-1\right)-\frac{\Delta S_{IS}}{\Delta T_{S}}T_{CS},$$

which leads to:

(43) $$T_{H}{}^{*}=\sqrt{\frac{T_{HSi\;}T_{CS\;}}{1+\frac{T_{CS\;}}{\Delta T_{S}}\frac{\Delta S_{IS}}{G_{H}}\;}}\;.$$

It is well known that internal irreversibility always decreases *W*. Hence, (41) is valuable by emphasizing in the denominator two factors that modify the optimal temperature T_H_*:

- the first, an intensive one: $\frac{T_{CS\;}}{T_{HSi}-\;T_{CS}}$;
- the second, an extensive one: $\frac{\Delta S_{IS}}{G_{H}}$.

The last factor is not only related to the converter through Δ*S_IS_*, but also to the hot source by the finite physical dimension *G_H_* (coupling).

This shows the interest of an entropic description of the system. Thus, the corresponding maximum power output is given by:

(44) $$\;\;MaxW=G_{H}{\left(\sqrt{T_{HSi}}-\sqrt{I\cdot T_{0\;}}\right)}^{2},$$

with

(45) $$I=\frac{T_{0\;}}{\Delta T_{S}}\cdot \frac{\Delta S_{IS}}{G_{H}}.$$

The associated first law efficiency is:

(46) $$\eta_{I}\left(MaxW\right)=\frac{{\left(1-\sqrt{\frac{I\cdot T_{0\;}}{T_{HSi}}}\right)}^{2}}{1-\sqrt{\frac{T_{0\;}}{I\cdot T_{HSi}}}},$$

### 3.3. Quasi-Chambadal Model with Added Heat Transfer Entropy Constraint and Production of Entropy

This optimization is based on (21) from which the temperature *T_H_* is expressed as:

(47) $$T_{H}=\frac{G_{H}}{G_{H}+\Delta S}T_{HS\;},$$

with Δ*S*, reference heat transfer entropy associated to the heat input. This result is an important one, since it illustrates the coupling between the source (*G_H_*) and the converter (Δ*S*).

The expression of the mechanical work *W* can be easily derived as a function of the newly chosen variable Δ*S*:

(48) $$W=\left[\frac{T_{HSi\;}G_{H}}{G_{H}+\Delta S}\;-T_{0\;}\right]\Delta S-T_{0\;}\Delta S_{I}.$$

This optimum with respect to Δ*S* corresponds to a converter adapted to the hot source. It results that *W* becomes maximum when Δ*S_I_* is related to Δ*S* (to be specified) by the following function:

(49) $$\frac{\partial W}{\partial \Delta S}=0={\left(\frac{G_{H}}{G_{H}+\Delta S}\right)}^{2}T_{HSi\;}-T_{0}-T_{0\;}\frac{\partial \Delta S_{I}}{\partial \Delta S}\;.$$

Following different assumptions, one can consider:

(i) the presence of the Novikov’s irreversibility degree [9], that is expressed by:

(50) $$\frac{\partial \Delta S_{I}}{\partial \Delta S}=d_{I}.$$

In this case, one gets at the optimum:

(51) $$\Delta S^{*}=G_{H}\left[\frac{\sqrt{T_{HSi\;}}}{\sqrt{T_{0\;}\left(1+d_{I}\right)}}-1\right].$$

It results that the optimum heat transfer entropy ΔS* diminishes with increasing irreversibility *d_I_*, and the same goes for *MaxW* and the cycle efficiency:

(52) $$MaxW=G_{H}{\left(\sqrt{T_{HSi\;}}-\sqrt{T_{0\;}\left(1+d_{I}\right)}\right)}^{2},$$

(53) $$\eta_{I}\left(MaxW\right)=1-\sqrt{\frac{T_{0\;}\left(1+d_{I}\right)}{T_{HSi}}},$$

where $d_{I}\leq \left(T_{HSi\;}-T_{0\;}\right)/T_{0\;}$.

(ii) when Δ*S_I_* is a constant parameter, one finds similarly:

(54) $$\Delta S^{*}=G_{H}\left[\frac{\sqrt{T_{HSi\;}}}{\sqrt{T_{0\;}}}-1\right],$$

and

(55) $$MaxW=G_{H}{\left(\sqrt{T_{HSi\;}}-\sqrt{T_{0\;}}\right)}^{2}-T_{0\;}\Delta S_{I},$$

(56) $$\eta_{I}\left(MaxW\right)=1-\sqrt{\frac{T_{0\;}}{T_{HSi}}}\left(1+\frac{\sqrt{T_{0\;}}}{\sqrt{T_{HSi\;}}-\sqrt{T_{0\;}}}\frac{\Delta S_{I}}{G_{H}}\right).$$

(iii) Δ*S_I_* may also depend of *T_H_* in a linear form, as in Section 3.2, case ii), namely:

(57) $$\Delta S_{I}=d_{IS}\frac{T_{H\;}-T_{0\;}}{T_{HSi\;}-T_{0\;}}\Delta S,$$

from which one gets:

(58) $$W=\left(T_{H\;}-T_{0\;}\right)\Delta S\left(1-T_{0\;}\frac{d_{IS}}{T_{HSi\;}-T_{0\;}}\right),$$

where $d_{IS}\leq \left(T_{HSi\;}-T_{0\;}\right)/T_{0\;}$ is the parameter of entropic proportionality corresponding to the irreversibility degree of Novikov [24], but relative to the system composed of a source and converter (the heat transfer from the converter to the sink being neglected in the model).

Knowing the expression of *T_H_* as a function of Δ*S*, one gets:

(59) $$W=\left(T_{HSi\;}\frac{G_{H}}{G_{H}+\Delta S}-T_{0\;}\right)\Delta S\left(1-T_{0\;}\frac{d_{IS}}{T_{HSi\;}-T_{0\;}}\right).$$

The optimum of the mechanical work with respect to Δ*S* is obtained after some calculations as:

(60) $$MaxW=G_{H}{\left(\sqrt{T_{HSi\;}}-\sqrt{T_{0\;}}\right)}^{2}\left(1-\frac{T_{0\;}}{T_{HSi\;}-T_{0\;}}d_{IS}\right).$$

The corresponding first law efficiency is:

(61) $$\eta_{I}\left(MaxW\right)=\left(1-\sqrt{\frac{T_{0\;}}{T_{HSi}}}\right)\left(1-\frac{T_{0\;}d_{IS}}{T_{HSi\;}-T_{0\;}}\right),$$

These two quantities are decreasing with the parameter *d_IS_*. Thus, the mechanical work *W* is diminished by the term $\left(1-T_{0\;}\frac{d_{I,S}}{T_{HSi\;}-T_{0\;}}\right)$, as shown in (59). Also, (61) clearly proves that irreversibility decreases the efficiency corresponding to maximum work.

## 4. Conclusions

This work presents a short history of the Carnot cycle approaches developed in the frame of Equilibrium Thermodynamics, of the actual endo-irreversible Carnot engine in Irreversible Thermodynamics, and for the Chambadal model.

The analytical model developed in the frame of Equilibrium Thermodynamics integrates the irreversibility in the natural form of an entropy production, or in an entropic ratio form (with various justified and correlated definitions) and is mainly related to the irreversibility degree introduced by Novikov [32]. 

The mechanical work of the irreversible cycle remains linked to the form of the irreversible isotherms and adiabatics, as has been shown in Section 2.1. The irreversibilities distribution between isothermal and adiabatic processes leads to framing the actual work between zero and max *MaxW*, with the corresponding efficiencies pertaining to the interval between zero and the Carnot efficiency.

For the Chambadal model, the cycle approach or the one in the stationary dynamic regime come down to the same form, by introducing a characteristic property *G_H_*, which is representative of the finite physical dimensions connected to the reference heat transfer entropy Δ*S*, characterizing the converter.

It results in the existence of an optimum of the mechanical work either in terms of intensive variable *T_H_*, or in terms of extensive variable Δ*S*.

The two approaches leading to coherent results, but with different interpretations, provide a new perspective on the optimization of the Carnot engine, mainly by coupling of the hot source with the converter (*G_H_* and Δ*S*).

Extensions are in progress, which are based on more complete models, particularly on the Curzon-Ahlborn one, that considers the non-equilibrium at source and sink. Also, the stochastic thermodynamics approach [33,34] is intended to be used in future works to evaluate different sources of irreversibility in the corresponding heat engines exactly, without additional assumptions. It could offer an alternative to evaluate the analytical results provided by our phenomenological approach.

## Abbreviations

*Symbols*	
*A*	heat transfer area, [m^2^]
*c_p_*	specific heat at constant pressure, [J/(kg K)]
$\dot{C}$	heat capacity rate, [W/K]
*d*	Novikov’s irreversibility degree, [-]
*ECOP*	ecological coefficient of performance, [-]
*G*	finite physical dimension, [J/K]
*K*	heat transfer conductance, [W/K]
*I*	entropic ratio, [-]
*NTU*	number of heat transfer units, [-]
*Q*	heat, [J]
$\dot{Q}$	heat transfer rate, [W]
S	entropy, [J/K]
T	temperature, [K]
U	overall heat transfer coefficient, [W/(m^2^ K)]
W	mechanical work, [J]
*Greek Symbols*	
*ε*	effectiveness, [-]
*η*	efficiency, [-]
*Subscripts*	
*C*	relative to the cycled fluid at the cold source
*Co*	relative to compression
*CS*	cold source or sink
*Ex*	relative to expansion
*H*	relative to the cycled fluid at the hot source
*HS*	hot source
*HSi*	hot source
*I*	irreversibility of the converter
*IS*	irreversibility of the system (source-converter-sink)
*S*	source or sink, relative to temperature difference of source and sink
*T*	total, relative to extreme temperature difference of the cycled fluid
*0*	ambient
